# A Rare Case of Phytobezoar Related to Occupational Exposure

**DOI:** 10.7759/cureus.54826

**Published:** 2024-02-24

**Authors:** Madalena Santos, Nuno R Carreira, Joana Gouveia, Alba Acabado

**Affiliations:** 1 Internal Medicine, Hospital Santa Maria, Centro Hospitalar Universitário Lisboa Norte, Lisboa, PRT; 2 Medicine 2, Hospital Santa Maria, Centro Hospitalar Universitário Lisboa Norte, Lisboa, PRT

**Keywords:** git endoscopy, foreign body, abdominal pain, ocuppational exposure, phytobezoars

## Abstract

Bezoars constitute a compacted collection of undigested or partially digested material, potentially leading to intestinal obstruction. They most frequently occur in the stomach, with classification based on their composition.

Many gastric bezoars are asymptomatic and frequently manifest in patients with gastrointestinal disturbances or psychiatric issues.

We present a rare case of a bezoar related to occupational exposure that illustrates the least-discussed health risks associated with certain jobs.

## Introduction

The term bezoar refers to objects believed to possess curative properties [1]. There are several types of bezoars, including phytobezoars, composed of indigestible fruits and vegetables; trichobezoars, containing hair; and pharmacobezoars, representing concretions of ingested drugs, especially sucralfate and aluminum hydroxide gel [2-4].

Several risk factors can be associated with the formation of bezoars, such as postoperative complications after gastric bypass, psychiatric disorders like hair consumption, and conditions such as diabetes mellitus and mixed connective tissue diseases that delay gastric emptying [5].

Most cases of bezoars cause no symptoms, but they can sometimes lead to abdominal pain, nausea, vomiting, postprandial fullness, and anorexia [2]. Unusually, there can be complications such as ileus and intestinal obstruction, gastrointestinal bleeding due to ulceration, intussusception, and peritonitis [3,4]. Considering that the symptoms are not specific to bezoars, the diagnosis can be challenging due to their low incidence [4].

We present a case report of an adult woman with bezoar - a rare condition, in this case, caused by occupational exposure.

## Case presentation

A 50-year-old woman was admitted to the emergency department with a history of acute abdominal pain located on the epigastric area and radiating to the lower quadrants. The pain started in the evening as colic pain that worsened after eating. The patient also mentioned nausea and postprandial fullness for at least two weeks. On physical examination, the patient had a fever, a moderately distended abdomen with active peristalsis, and tenderness upon deep palpation of the epigastric area, right and left lower quadrants, without signs of peritoneal inflammation, and negative Murphy and Blumberg signs. The laboratory evaluation showed normocytic, normochromic anemia and elevated inflammatory parameters without significant changes in the urinalysis (Tables 1-2). The ECG revealed a sinus rhythm, heart rate of 96 bpm, without any abnormalities. 

**Table 1:** Laboratory results.

Laboratory evaluation	Results	Normal range	Unit
Hemoglobin	11.3	12.0-15.3	g/dL
Mean corpuscular volume	90.8	80.0-97.0	fL
Mean corpuscular hemoglobin	34.5	31.5-35.5	g/dL
Leucocytes	16.9	4.0-11.0	x10^9 ^L^-1^
Neutrophils	15.2	1.9-7.5	x10^9 ^L^-1^
Platelets	430	150-450	x10^9 ^L^-1^
C-reactive protein	5.91	<0.5	mg/dL
Serum creatinine	0.62	0.50-0.90	mg/dL
Blood urea nitrogen	23	16-49	mg/dL
Aspartate transaminase	16	0-32	U/L
Alanine aminotransferase	13	0-33	U/L
Lactate dehydrogenase	254	100-250	U/L

**Table 2:** Urinalysis results.

Urinalysis	Results	Unit
Nitrates	Negative	mg/dL
Proteins	Negative	mg/dL
Albumin	30 (+)	mg/L
Leukocytes	Negative	cells/µL
Erythrocytes	80 (++)	cells/µL

**Figure 1:**
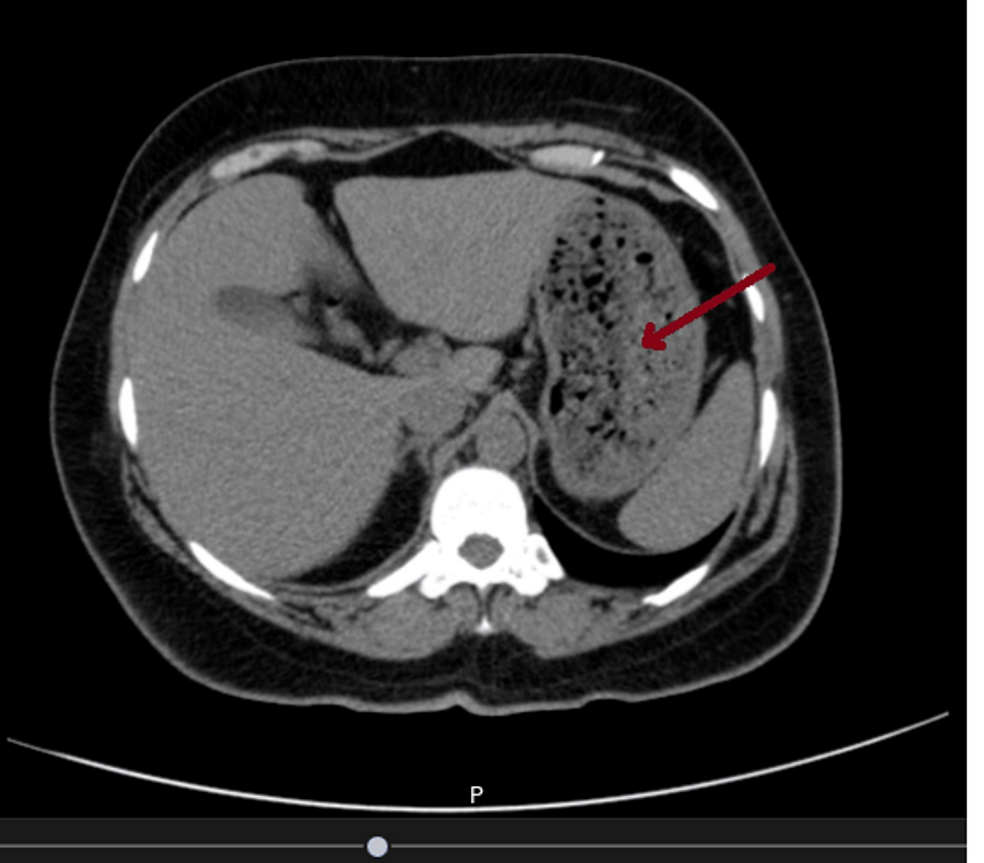
CT scan image showing the patient's stomach filled with material resembling a bezoar.

The patient underwent an abdominal CT scan that revealed a significant amount of material in the stomach resembling a bezoar (Figure 1).

**Figure 2:**
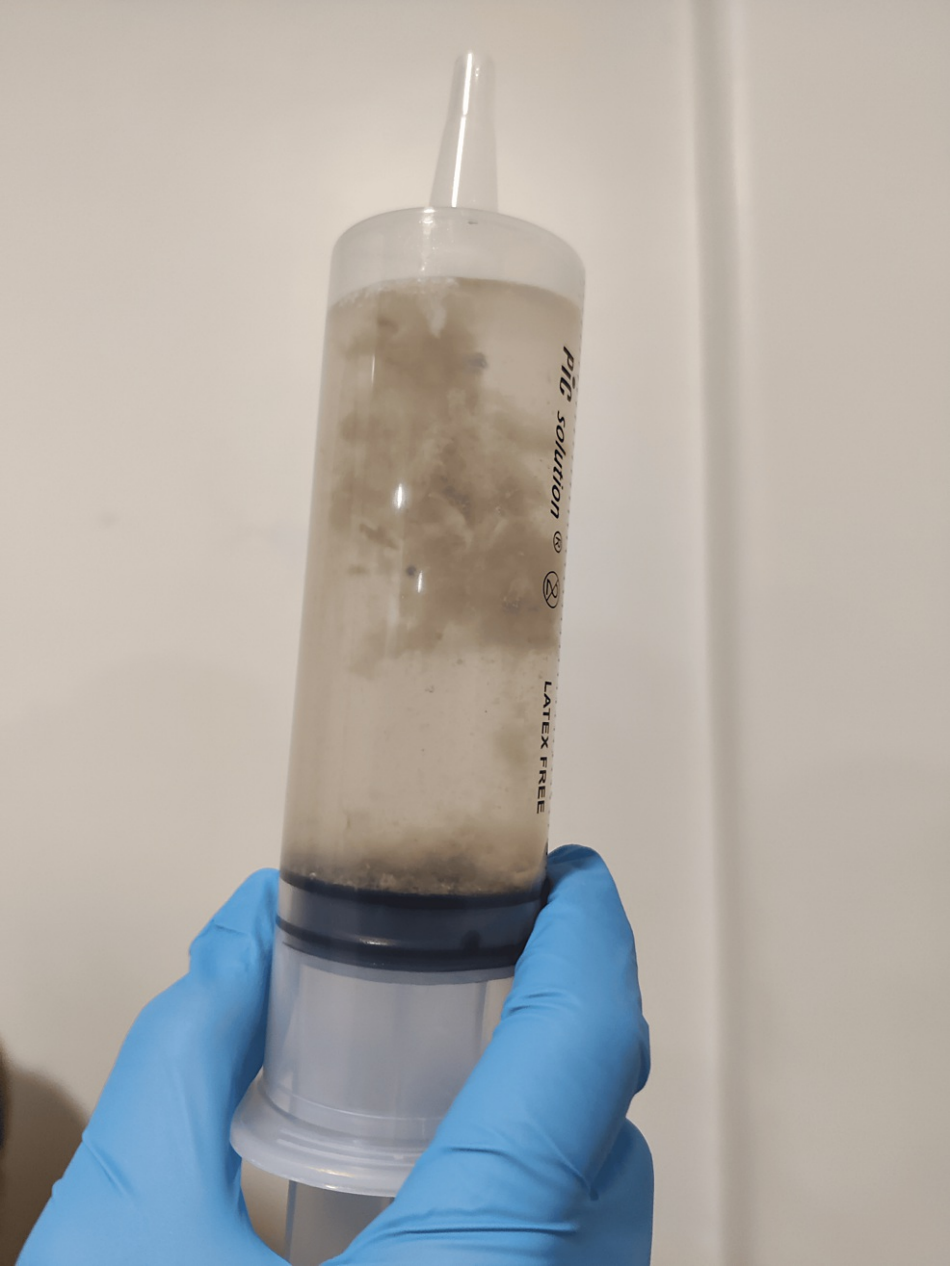
Syringe filled with digestive content from the patient. Image credit: Madalena Santos.

**Figure 3:**
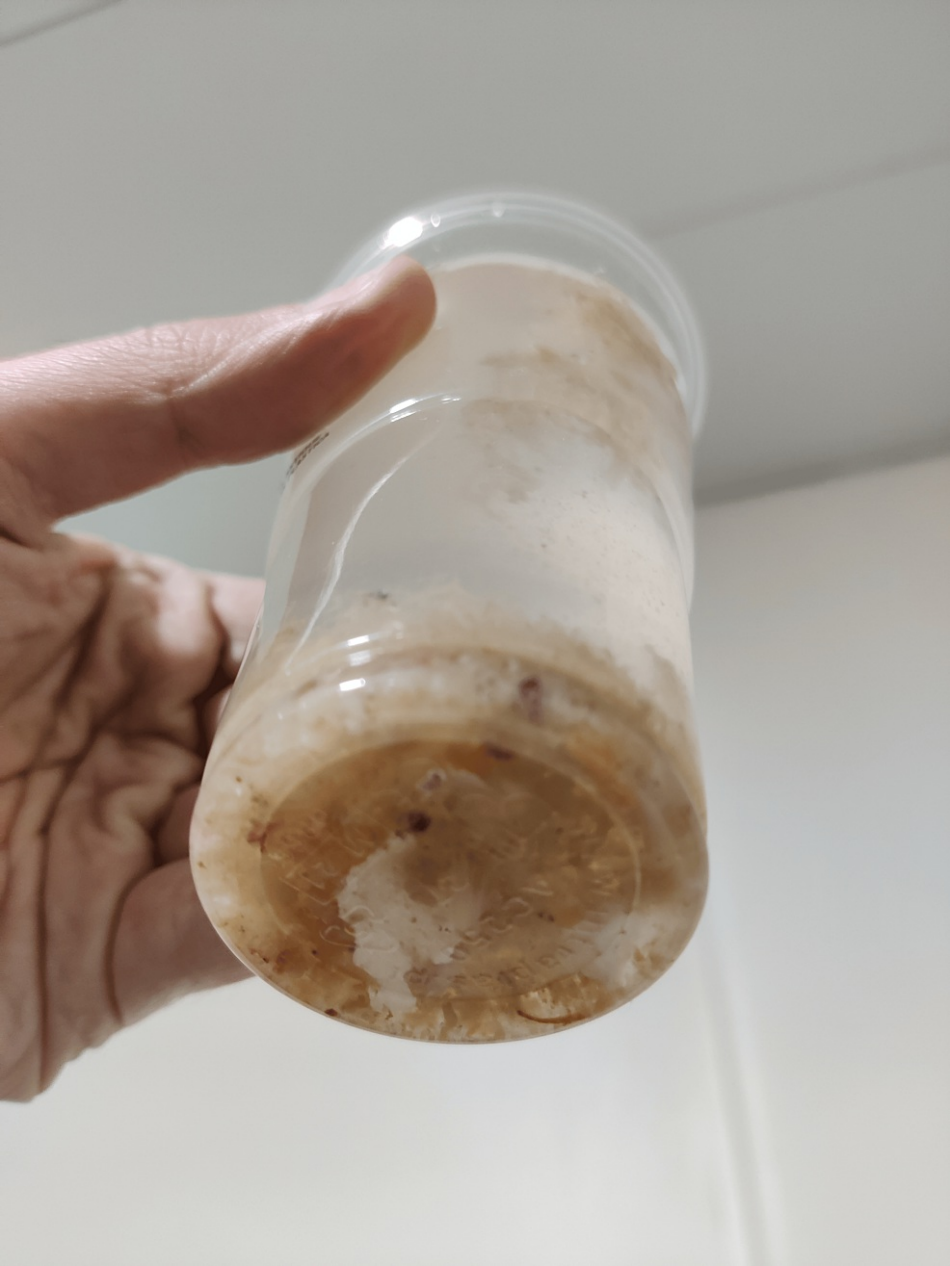
Glass filled with digestive content from the patient. The mixture of dust particles and nail debris that was removed from the patient filled up to four glasses of water. Image credit: Madalena Santos.

Considering the aforementioned findings, a nasogastric tube was placed, and a large amount of dust particles and nail debris mixed with food were manually aspirated, providing immediate symptomatic relief (Figures 2-3).

It was later discovered that the patient had worked as an aesthetician for about 30 years, frequently exposed to smoke, nails, and acrylic particles without using protective equipment, thus inhaling those smoke and dust particles. The patient also mentioned a habit of biting her own nails.

Afterward, she underwent a gastrointestinal endoscopy that removed the remaining dust particles and nail debris, and the procedure proceeded without any complications.

A psychiatric evaluation was conducted in the emergency room, and the patient confirmed chronic nail biting since childhood, which worsened after she started working as an aesthetician.

The patient was later discharged in clinically stable condition with instructions to monitor abdominal pain, nausea, vomiting, or any other suspicious symptoms. The recommendation included using protective equipment during work and seeking follow-up in a psychiatry consultation.

## Discussion

Most foreign bodies can be present in the stomach without causing any symptoms and can be an incidental finding on imaging tests. Most foreign bodies of different sizes can be swallowed and pass through the gastrointestinal tract undetected^ ^[6].

Regarding phytobezoar obstruction, it occurs most frequently in the jejunum and/or ileum^ ^[7].

In our case report, the symptoms were caused by a phytobezoar composed of nails, dust particles, and food, which had accumulated in the stomach of a patient exposed to nail particles for years.

Since the patient used to bite her nails and was exposed to smoke and acrylic particles without protective equipment for years, there was an accumulation of those substances with food in her stomach, causing mild symptoms. 

Similar to other cases reported in the literature involving bezoars, the patient was asymptomatic for years and developed mild symptoms.

The approach to phytobezoars can involve chemical dissolution, endoscopic removal, adjuvant prokinetics, and surgery [8].

If an acute intestinal obstruction is caused by bezoars, immediate surgical intervention may be necessary. However, if the bezoar is located in the esophagus or stomach, conservative treatment could be attempted first [4].

In our patient, the initial approach involved manually removing the material from the stomach, followed by a gastrointestinal endoscopy to extract the remaining particles and rule out other complications. 

Fortunately, most of the undigested content was successfully removed manually and the gastrointestinal endoscopy revealed no abnormal findings, besides a small amount of nail and dust particles, similar to the previous pictures. 

Regarding the fever and increase in inflammatory parameters, they were considered nonspecific, as the phytobezoar did not lead to any major complications, such as septic shock secondary to perforation.

## Conclusions

There are a few cases of occupational exposure-related bezoars. Nonetheless, the importance of using adequate equipment remains. 

Phytobezoars, although rare, can cause significant complications that may necessitate immediate surgical intervention.

Therefore, it is necessary to recognize the main symptoms related to their development, request the necessary imaging techniques, and make an accurate diagnosis to prevent further organ damage, particularly in certain occupations.
